# Infantile Epileptic Spasm Syndrome Caused by *TAF1* Missense Variants: Expanding the Epileptic Phenotype and Revealing Underlying Impairments in Neuronal Excitability and Development

**DOI:** 10.1155/humu/4104648

**Published:** 2026-09-16

**Authors:** Hui Xiao, Changning Xie, Pan Peng, Xiaoyuan Ni, Leilei Mao, Jing Peng

**Affiliations:** ^1^ Department of Pediatrics, Xiangya Hospital, Central South University, Changsha, Hunan, China, csu.edu.cn

**Keywords:** infantile epileptic spasms syndrome, loss-of-function, neuronal development, neuronal excitability, *TAF1*

## Abstract

**Background:**

Pathogenic variants in *TAF1* have previously been associated with X‐linked intellectual disability and X‐linked dystonia‐parkinsonism. Epilepsy is observed in approximately 24.4% of affected individuals, ranging from focal seizures to generalized seizures including either tonic‐clonic, tonic, myoclonic, atonic or absences. To date, no cases of infantile epileptic spasm syndrome (IESS) have been associated with this neurogenetic condition. In this study, we are aimed at investigating the association between *TAF1* and IESS.

**Methods:**

We performed whole‐exome sequencing in two unrelated Chinese patients with IESS. Functional consequences were assessed using in vitro overexpression assays, transcriptome profiling of Neuro‐2a cells with stable *TAF1* knockdown, morphological, and electrophysiological analyses in primary mouse cortical neurons.

**Results:**

We identified two novel *TAF1* missense variants (c.236C > A, p.T79N; c.4771G > A, p.D1591N) in the two patients. Both novel *TAF1* missense variants led to reduced TAF1 protein expression, suggesting a loss‐of‐function mechanism. Transcriptome analysis revealed that *TAF1* deficiency resulted in significant downregulation of key neuronal genes involved in axon guidance and epileptogenesis, including *Kcnn2*, *Kcna4*, *Draxin*, and *Lgi1*. In primary mouse cortical neurons, *TAF1* knockdown markedly impaired neurite outgrowth and increased neuronal excitability.

**Conclusion:**

Our findings establish *TAF1* missense variants as a novel cause of IESS—a previously unrecognized phenotype—thereby expanding the epileptic phenotype of *TAF1*‐related disorders. Furthermore, we demonstrate that *TAF1* deficiency contributes to disease pathogenesis by impairing neuronal development and increasing neuronal excitability, likely through the downregulation of *Kcnn2*, *Kcna4*, *Draxin*, and *Lgi1*.

## 1. Introduction

Infantile epileptic spasm syndrome (IESS) represents one of the most devastating forms of developmental and epileptic encephalopathy, clinically defined by the triad of epileptic spasms with age‐dependent onset, interictal hypsarrhythmia on electroencephalogram (EEG), and arrested or regressed neurodevelopment [1]. Genetic factors constitute a predominant etiology; variants in over 70 genes have been implicated through next‐generation sequencing of large patient cohorts [1].

The *TAF1* gene (Xq13.1) encodes the largest subunit of transcription factor IID (TFIID), an essential multiprotein complex that nucleates RNA polymerase II preinitiation complex assembly and directs transcription initiation at most eukaryotic promoters [2, 3]. The critical developmental role of TAF1 was first illuminated in disease contexts through two seminal discoveries. Makino et al. [4] identified SVA retrotransposon insertion in intron 32 of *TAF1* as the cause of X‐linked dystonia‐parkinsonism (XDP) (DYT3), demonstrating striatum‐specific reduction of TAF1 and its neuron‐specific isoform. Subsequently, O′Rawe et al. [5] established *TAF1* missense variants as a cause of X‐linked intellectual disability (XLID), reporting 14 affected males from 11 families with characteristic dysmorphic features and cognitive‐motor impairment. Their follow‐up study in 2020 expanded this cohort to 41 individuals, among whom 10 (24.4%) exhibited epilepsy—encompassing focal, generalized tonic‐clonic, tonic, myoclonic, atonic, and absence seizures [6]. Notably, no IESS cases had been documented in this genetic spectrum.

The neurodevelopmental functions of TAF1 extend beyond transcriptional initiation. Gudmundsson et al. [7] demonstrated that TAF1 deficiency in zebrafish disrupts embryogenesis and perturbs neurodevelopmental programs, whereas Janakiraman et al. [8] reported that postnatal CRISPR/Cas9‐mediated Taf1 knockout in rat cerebellum yields Purkinje cell loss, cortical morphological abnormalities, and aberrant excitatory neurotransmission—though this model paradoxically lacked the intellectual disability, microcephaly, or seizures characteristic of human disease. This discrepancy likely reflects the fundamental limitation of postnatal gene targeting: the proliferation, migration, and differentiation of neural progenitors, together with forebrain patterning, occur predominantly during embryonic stages that this neonatal model cannot recapitulate [7]. Consequently, no validated animal model currently exists for TAF1‐related encephalopathy, and the mechanisms linking TAF1 variants to epileptogenesis remain unexplored.

Here, we report two unrelated male probands with IESS harboring novel maternal TAF1 missense variants—c.236C > A (p.T79N) and c.4771G > A (p.D1591N)—both associated with severe epileptic spasms and profound developmental impairment. Through integrated functional analyses, we demonstrate that these variants reduce TAF1 protein stability, identify downstream transcriptional targets critical for neuronal development and excitability, and establish cellular mechanisms of impaired neurite outgrowth and hyperexcitability. Our findings define IESS as a previously unrecognized phenotypic consequence of TAF1 pathogenic variants, extending the clinical and mechanistic spectrum of TAF1‐related disorders.

## 2. Methods

A preliminary version of this work was previously published as a preprint [9].

### 2.1. Subjects

The subjects were two Chinese cases with infantile spasms syndrome carrying *TAF1* variants. Their genetic variants, brain magnetic resonance imaging (MRI), EEG, and clinical manifestations were reviewed. Detailed developmental milestones before and after seizure onset, serial EEG recordings before and after treatment, antiseizure medication regimens (agents, dosages, and courses), quantitative seizure frequency (daily spasm counts and clustering), and dysmorphic or movement‐disorder features were systematically collected from medical records and caregiver interviews. We obtained written informed consent from the parents of patients. The institutional review boards at Central South University Xiangya Hospital approved this study. We conducted whole‐exome sequence (WES) of family trios by extracting genomic DNA from the peripheral blood of the probands and parents.

### 2.2. Plasmid Constructs

A pcDNA3.1 vector carrying TAF1(NM_004606.4) with a tag GFP at the 3 ^′^ end was obtained from GeneScript (Nanjing, China). The TAF1 variants were produced via PCR using KOD‐Plus High‐Fidelity DNA polymerase (Toyobo, Osaka, Japan), followed by DpnI enzyme (Biolegend, San Diego, California, United States). Short hairpin RNA (shRNA) was designed to knock down mouse *Taf1* by targeting the following sequence of *Taf1*: 5 ^′^‐GGAAGGAACTCCAGCGGATGC‐3 ^′^. shRNA was cloned into the FUGW lentiviral vector, and lentivirus was provided by Genechem Co., Ltd. (Shanghai, China). The GFP plasmid was purchased from GeneScript (Nanjing, China).

### 2.3. Cell Culture, Transfections, and Transduction

HEK293T cells were maintained in Dulbecco′s Modified Eagle′s medium (DMEM) (Gibco) containing 10% fetal bovine serum (FBS) (Gibco) at 37°C. Neuro‐2a cells were kept in DMEM‐F12 medium with 10% FBS. We transduced mouse neuroblastoma Neuro‐2a cells with lentiviral particles expressing TAF1‐targeting shRNA to generate stable TAF1‐knockdown Neuro‐2a cells. Transduced cells were selected with puromycin (2 *μ*g/mL for 7–14 days), and Western blotting was conducted to confirm knockdown efficiency. The mouse cortical neurons are separated from E16 C57BL/6 mice and cultured in plating medium (minimum essential medium [MEM] + 10*%*FBS; Gibco, Carlsbad, California) on coverslips coated with poly‐d‐lysine (0.1 mg/mL; BD Biosciences, San Jose, California, United States). The seeding density per well of the 24‐well plate ranged from 5 × 10^4^ to 20 × 10^4^ cells. The plating medium was replaced by culture medium (neurobasal medium + B27 + GlutaMAX; Invitrogen, Carlsbad, California, United States).

The constructs were transfected into Neuro‐2a and HEK293T cells using Lipofectamine 3000 reagent (Invitrogen). On Day 1 in vitro (DIV 1), E16 mouse cortical neurons were cotransfected with TAF1 shRNA and GFP‐expressing plasmid (5:1 ratio) using Lipofectamine 3000. The GFP reporter was used to visualize neuronal morphology. After 4 days, the neurons were fixed to evaluate the neurite length. At DIV5, the neurons were transfected with plasmids or shRNA using calcium phosphate (Beyotime, China) and fixed at DIV18 for dendritic spine analyses.

### 2.4. Immunofluorescence

After washing with phosphate buffer solution (PBS) three times, the neurons were fixed in 4% paraformaldehyde (PFA) for 15 min. The fixed neurons were permeabilized with 0.1% Triton X‐100 in PBS for 10 min and blocked with 0.1% Triton X‐100 PBS buffer (PBST) containing 10% bovine serum albumin at room temperature for 30 min. The neurons were incubated with primary antibodies at 4°C overnight, washed with sterile PBS three times, and then incubated with secondary antibodies at room temperature for 1 h.

Neuronal morphology was evaluated using a Leica TCS SP5 II confocal system (Leica Microsystems). Confocal images for neurite outgrowth were acquired using a 20x dry objective, and dendritic spines were imaged using an oil immersion 100x objective. Each confocal image of a dendritic spine was a *z*‐stack of approximately 5–15 sections (0.3‐*μ*m step size) to ensure complete 3D reconstruction. Cells expressing GFP were randomly selected for morphometric assessment to examine neurite length and dendritic spine density in cultured primary cortical neurons. Neurite outgrowth and dendritic spines were measured using ImageJ software (https://imagej.nih.gov/ij/download.html). Sixty neurons across three independent experiments were counted to analyze axonal length and total neurite length. Spine density was calculated as spines/10‐*μ*m dendritic length. Classification of dendritic spines was conducted on maximum‐intensity *z*‐projections. Fifteen neurons across three independent experiments were counted to analyze spine density and spine classification.

### 2.5. Transcriptome Sequencing and Analysis

Total RNA was extracted from stable TAF1‐knockdown Neuro‐2a cells. Stranded RNA sequencing libraries were prepared using the KC‐Digital Stranded mRNA Library Prep Kit for Illumina (DR08502, Seqhealth, China) following the manufacturer′s protocol. Library products (200–500 bp) were size‐selected, quantified, and sequenced on a DNBSEQ‐T7 platform (MGI Tech, China) in 150‐bp paired‐end mode. All sequencing procedures were conducted by Seqhealth Technology Co., Ltd (Wuhan, China). Reads were filtered using Trimmomatic (v0.36) to remove low‐quality sequences (*Q* < 20) and adapter contamination. PCR duplicates were eliminated using in‐house scripts to minimize the risk of amplification bias. Clean reads were mapped to the Mus musculus reference genome (Ensembl GRCm39) using STAR (v2.5.3a) using default parameters. Exon‐mapped reads were counted using featureCounts (Subread‐1.5.1) and normalized as RPKM values. The deduplicated consensus sequences were used for standard RNA‐seq analysis. They were mapped to the reference genome of mouse from Ensembl_GRCm39_111 (https://ftp. http://ensembl.org/pub/release-111/fasta/mus_musculus) using STAR software (Version 2.5.3a) with default parameters. Differential expression analysis was conducted using edgeR (v3.12.1) with thresholds of |fold − change| ≥ 2 and adjusted *p* value < 0.05. Gene Ontology (GO) analysis and Kyoto Encyclopedia of Genes and Genomes (KEGG) enrichment analysis for differentially expressed genes were conducted using KOBAS software (Version: 2.1.1) with a *p* value cutoff of 0.05 to judge significant enrichment. Alternative splicing events were detected using rMATS (Version 3.2.5) with an FDR value cutoff of 0.05 and an absolute value of *Δ* *ψ* of 0.05.

### 2.6. Western Blotting

*Cells were transfected with shRNA or normal control, as well as point variants or WT TAF1 vectors. Total protein was rapidly extracted from cultured cells via RIPA lysis buffer supplemented with protease inhibitor cocktail solution (Millipore, Boston, Massachusetts, United States). Proteins were separated using 8% SDS-PAGE and transferred onto polyvinylidene fluoride membranes. The membranes were then incubated with primary antibodies at 4°C overnight, followed by incubation with secondary antibodies for 1 h at room temperature. Finally, the protein bands were visualized using Immobilon Western Chemiluminescent HRP Substrate (Millipore, Boston, Massachusetts, United States).*

### 2.7. qPCR

Total RNA was extracted from cells using the TRIzol reagent (Sigma‐Aldrich) following the manufacturer′s protocol, and then reverse‐transcribed into cDNA using HiScript II Q RT SuperMix for qPCR Kit (Vazyme, China). Then, cDNA was employed to conduct the quantitative reverse transcription‐PCR (qRT‐PCR) using a ChamQ Universal SYBR qPCR Master Mix kit (Vazyme, China) via the ABI QuantStudio 1 Flex instrument (Thermo Fisher).

### 2.8. Whole‐Cell Patch Clamp

Primary cortical neurons were transfected with TAF1‐targeting shRNA at 7 days in vitro (DIV 7), and whole‐cell patch‐clamp recording was conducted 1 week after transfection (DIV 14). All recordings were conducted at room temperature (22°C–24°C) using borosilicate glass pipettes (3–5 M*Ω* resistance) filled with intracellular solution containing (in mM): 123 K‐gluconate, 10 KCl, 0.1 CaCl_2_, 1 MgCl_2_, 1 EGTA, 0.2 Na‐GTP, 1.5 Mg‐ATP, 4 D‐glucose, and 10 HEPES (pH 7.3 adjusted with KOH, 290 mOsm). Extracellular artificial cerebrospinal fluid (aCSF) comprised 140 mM NaCl, 3 mM KCl, 2 mM CaCl_2_, 1 mM MgCl_2_, 10 mM HEPES, and 10 mM D‐glucose (pH 7.4 with NaOH, 310 mOsm). Action potentials were evoked through somatic current injection using 500‐ms depolarizing pulses delivered in 10‐pA increments (0–180‐pA range) at 5‐s intervals. Current injections typically elicited membrane potential depolarizations of ∼10 mV per step. Signals were amplified using a Multiclamp 700B amplifier (Molecular Devices), and low‐pass signals were filtered at 2 kHz and digitized at 10 kHz. Data acquisition and analysis were conducted using pCLAMP 10.7 software (Clampex for recording and Clampfit for analysis). The liquid junction potential was corrected offline. Recordings were obtained from 10 neurons per group across three independent experiments.

## 3. Data Analysis

GRAPHPAD/Prism 7.0 Software was utilized for statistical analysis (San Diego, California, United States). Student′s *t*‐test was applied to compare groups. All data are presented as mean ± standard error of the mean (SEM). The *p* value was divided into three levels:  ^∗^ *p* < 0.05,  ^∗∗^ *p* < 0.005, and  ^∗∗∗∗^ *p* < 0.0001. In the revised figures, exact *p* values are reported for each statistical comparison in addition to asterisk notation.

## 4. Results

### 4.1. Clinical Features of the Two Cases

Case 1 was a boy aged 11 years with seizure onset at 7 months of age. A maternal missense variant c.236 C > A (p. T79N) was identified in *TAF1.* Segregation studies revealed that the mother carried the same *TAF1* variant. His big brother and parents were all healthy. The perinatal history and family history were unremarkable. Before seizure onset, his psychomotor development was age‐appropriate: He could hold his head steadily at approximately 3 months and sit with support at 6 months. After the onset of spasms at 7 months of age, developmental progression stagnated. The spasms occurred frequently every day, typically in clusters of 5–8, with approximately 2–3 clusters per day. EEG showed typical hypsarrhythmia. The patient was diagnosed with IESS and treated with adrenocorticotropic hormone (ACTH) (2 U/kg/day for 28 days), valproic acid (VPA) (30 mg/kg/day) and topiramate (TPM) (2 mg/kg/day). Following ACTH treatment, follow‐up EEG at approximately 9 months of age showed resolution of hypsarrhythmia, although epileptiform abnormalities persisted. The patient continued to have occasional spasms (2–3 per day) despite the resolution of hypsarrhythmia. After adjustment of antiseizure medications, he became seizure‐free at 2 years of age, and follow‐up EEG at 2 years showed abnormal. Developmental progress remained slow; at 11 years of age, he could walk independently but not run, and could only speak in 7–8‐word sentences. Gesell Developmental Schedules scores showed moderate intellectual disability at 5 years of age. Brain MRI and blood and urine metabolic screening were normal.

Case 2, a 12‐year‐old boy, carried a maternal missense variant c.4771G > A (p. D1591N) in the *TAF1* gene. He was the only child in the family with healthy parents. The perinatal and family histories were unremarkable. His epileptic spasms started at 3 months of age. Before seizure onset, his development was delayed: he could not hold his head steadily at 3 months of age. Following onset, he exhibited developmental arrest and stagnation. The spasms occurred in clusters, approximately 3–5 clusters per day, each consisting of 10–50 individual spasms. At 10 months of age, he was evaluated at our hospital; EEG demonstrated typical hypsarrhythmia with spasms, and IESS was diagnosed. He was treated with ACTH (2 U/kg/day for 28 days), which was ineffective. Levetiracetam (LEV), VPA, and lamotrigine (LTG) were also ineffective. At 1.5 years of age, a ketogenic diet was initiated, achieving 6 months of seizure freedom, but spasms subsequently recurred. Thereafter, multiple adjustments of antiseizure medications were ineffective. At 3 years of age, his head circumference was 47 cm; he exhibited thin upper lip vermilion and severe developmental delay. He could hold his neck at 1.5 years of age and sit without support at 4.5 years of age. He exhibited severe global developmental delay with no language ability and was bedridden, with severe intellectual disability and hypotonia. Brain MRI and blood and urine metabolic screening were normal.

### 4.2. Molecular Findings

**Figure 1:**
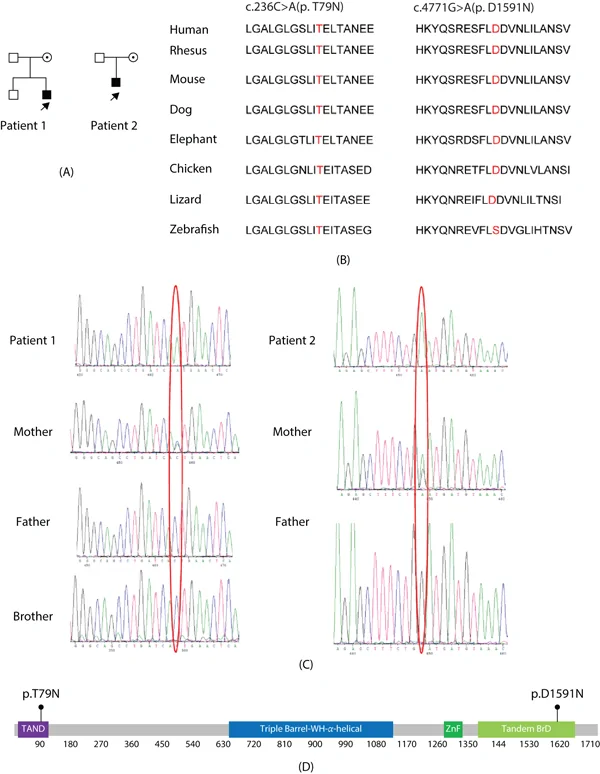
Evolutionary conservation of the two novel *TAF1* missense variants and the functional domains of the TAF1 protein. (A,B) Pedigrees and Sanger sequencing chromatograms showing that Patient 1 harbored a hemizygous c.236C > A variant and Patient 2 harbored a hemizygous c.4771G > A variant in *TAF1*, both inherited from their unaffected mothers. (C) Cross‐species protein sequence alignment showing high conservation of the mutated amino acid residues p.T79 and p.D1591 in TAF1. (D) Schematic of the five functional domains of TAF1; the domain previously labeled “Znk” is renamed ZnF (zinc‐finger domain), and the amino acid positions of each domain are now indicated. The two missense variants are located in the TBP‐binding domain and the double bromodomain, respectively. In silico prediction results are summarized in Table S1. ZnF, zinc finger.

Using trio‐based WES, we identified two novel TAF1 variants (c.236C > A, > A, p.T79N and c.4771G > A, > A, p.D1591N) in two unrelated Chinese individuals. No other pathogenic variants associated with ID or epilepsy were detected. Both variants were inherited from their asymptomatic mothers and confirmed by Sanger sequencing (Figure 1A,B). Evolutionarily, both variants affect residues that are highly conserved across diverse species (Figure 1C). Specifically, the p.T79N variant affects a highly conserved threonine residue within the TATA‐binding protein (TBP)–interacting domain, whereas the p.D1591N variant substitutes an evolutionarily conserved aspartate residue in the tandem bromodomain (Figure 1D). Neither variant was found in the ESP6500, 1000 Genomes or ExAC databases. The variant p.D1591N was present in one hemizygous male in the gnomAD database (0.0011%, gnomAD v.2.1.1). No phenotypic information is available for this carrier because gnomAD does not provide individual‐level clinical data; notably, a single observation in a population database does not exclude pathogenicity for a severe, early‐onset X‐linked disorder. The p.T79N variant was predicted to be deleterious by multiple prediction tools (CADD, PolyPhen2, Mutation taster, and LRT). The p.D1591N variant was predicted to be damaging by CADD, Mutation taster, and LRT, but benign by PolyPhen2 (Table S1). The in silico prediction results of both variants are summarized in Table S1.

### 4.3. The Protein Expression Levels of the Two *TAF1* Missense Variants Were Reduced

**Figure 2:**
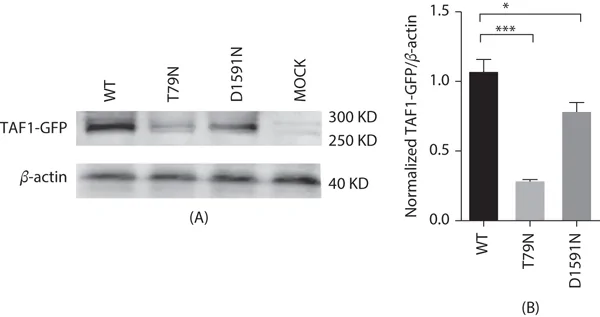
The effect of mutant TAF1 on the protein level. The constructed plasmids with the two novel *TAF1* missense variants tagged with GFP protein, transfected into HEK293T cells, reduced the protein levels of TAF1‐GFP. The GFP antibody (Invitrogen, Rabbit 1:1000) was used for this experiment. (A) Representative Western blots. (B) Quantification of TAF1 protein levels; the ordinate indicates the relative TAF1 protein level normalized to the WT group (set to 1.0). Exact *p* values are indicated for each comparison ( ^∗^ *p* < 0.05;  ^∗∗∗^ *p* < 0.001). GFP, green fluorescent protein; WT, wild‐type.

We used Western blotting to measure the protein levels of the missense variants of *TAF1* after overexpressing GFP‐tagged wild‐type or mutant TAF1 plasmids in HEK293T cells. We observed that the two variants resulted in significantly decreased expression levels of TAF1 (Figure 2A). The p.T79N variant led to a greater decrease in the expression level than the p.D1591N variant (Figure 2B).

TAF1 is a large protein, and its complete crystal structure is currently unavailable. We modeled these two sites of TAF1 against known crystal structures to clarify the impact of mutations on protein function. The results showed that p.D1591N is more likely to alter the DNA‐binding properties of TAF1 (see Figure S1 for details).

### 4.4. Transcriptome Analysis Revealed That *TAF1* Plays a Critical Role in Neurodevelopment Processes

**Figure 3:**
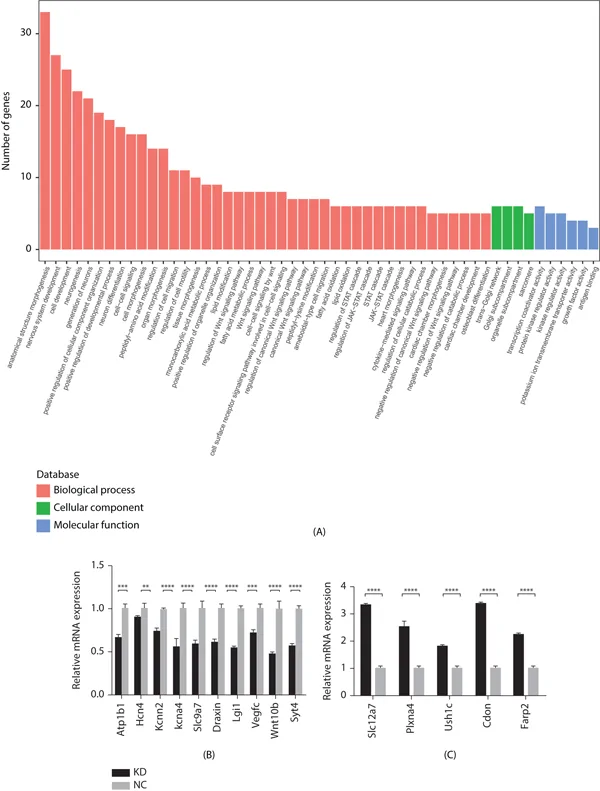
Differential expression analysis of RNA‐seq data from *Taf1* KD and control Neuro‐2a cells. (A) Gene Ontology (GO) enrichment analysis of downregulated and upregulated differentially expressed genes; the abscissa indicates −log10 (*p* value) and the ordinate lists the enriched GO terms. (B,C) Validation of selected upregulated or downregulated neurodevelopment‐related genes and ion channel genes. Exact *p* values are indicated for each comparison ( ^∗∗^ *p* < 0.05;  ^∗∗∗^ *p* < 0.001;  ^∗∗∗∗^ *p* < 0.0001). GO, Gene Ontology; KD, knockdown.

TAF1 is critical for transcription initiation. Therefore, it is reasonable to assume that variants of *TAF1* may alter gene expression. Accordingly, we generated stable TAF1 knockdown cell lines using lentiviral delivery of shRNA, followed by RNA sequencing of both TAF1‐depleted Neuro‐2a cells and control samples for differential gene expression analysis (Figure S2). GO enrichment analysis of differentially expressed genes indicated that the significantly enriched cellular components were associated with the development of the nervous system, neurogenesis, and neuron generation (Figure 3A). Data revealed that TAF1 is important for neuronal development. We next validated changes in the expression of selected neurodevelopment‐related genes based on qPCR. Most genes (15/20, 75%) displayed similar expression fold changes. In TAF1‐depleted Neuro‐2a cells, *Slc12a7*, *Plxna4*, *Ush1c*, *Cdon*, and *Farp2* were significantly upregulated, whereas *Atp1b1*, *Hcn4*, *Kcnn2*, *Kcna4*, *Slc9a7*, *Draxin*, *Lgi1*, *Vegfc*, *Wnt10b*, and *Syt4* were significantly downregulated compared with controls (Figure 3B,C). Notably, the expression levels of potassium ion genes *Kcnn2* and *Kcna4* were decreased.

### 4.5. *TAF1* Downregulation Led to Neurite Outgrowth Defect in Cultured Cortical Neurons

**Figure 4:**
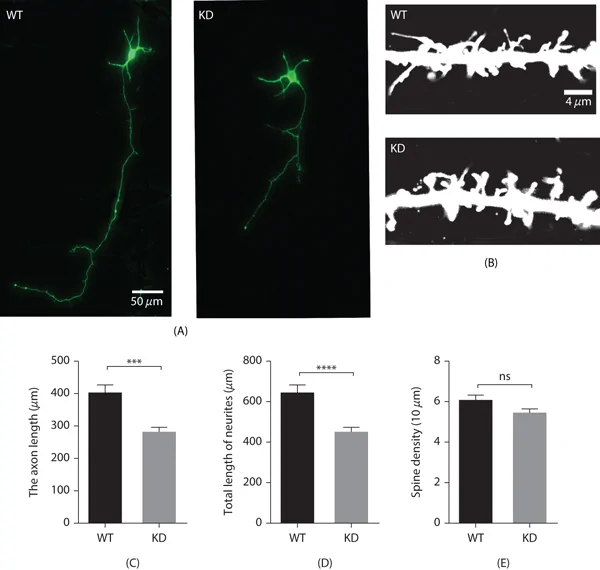
Effects on neurite outgrowth and dendrite development in *Taf1* KD neurons. (A,C,D) Primary mouse cortical neurons cotransfected with shRNA or control vector with the plasmid encoding GFP were incubated with DAPI and GFP antibody (Invitrogen, Rabbit 1:500) to visualize the overall neuronal morphology. Taf1 KD neurons exhibited reduced axon length and total neurite length, which included both axonal and dendritic processes. Scale bar, 50 *μ*m. (Three independent experiments, *n* = 60). (B,E) Taf1 knockdown did not affect dendrite spine density. Scale bar, 4 *μ*m. (Three independent experiments, *n* = 15). Exact *p* values are indicated for each comparison ( ^∗∗∗^ *p* < 0.001;  ^∗∗∗∗^ *p* < 0.0001). DAPI, 4 ^′^,6‐diamidino‐2‐phenylindole; GFP, green fluorescent protein; KD, knockdown; shRNA, short hairpin RNA.

We cotransfected primary mouse embryonic cortical neurons with TAF1‐targeting shRNA and a GFP reporter plasmid to investigate the role of TAF1 in neuronal development. This approach allowed us to simultaneously knock down TAF1 expression and visualize neuronal morphology. The results indicated that the number of axons and total neurites of GFP‐labeled neurons treated with *Taf1* shRNA were significantly decreased compared with control neurons (Figure 4A,C,D). Therefore, we measured the densities of dendritic spines in neurons treated with TAF1 shRNA. Downregulation of TAF1 did not alter the number of primary dendritic spines compared with control neurons (Figure 4B,E). Together, these data showed that *TAF1* is needed for the development of neurites but has no specific effect on dendritic spines.

### 4.6. *TAF1* Downregulation Increases Cell Excitability in Cultured Cortical Neurons

**Figure 5:**
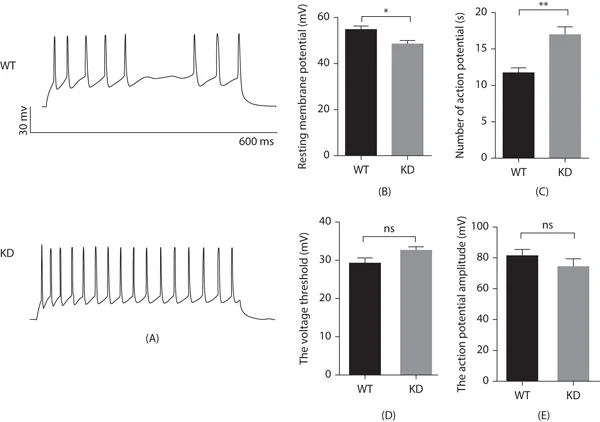
Electrophysiological abnormalities caused by *Taf1* knockdown. (A,B,C) Whole‐cell patch clamp recording of Taf1 knockdown and control neurons showed that the resting membrane potential was low and the number of action potentials increased in Taf1 KD neurons. (D,E) The voltage threshold and the action potential amplitude were not altered by Taf1 knockdown. (Three independent experiments, *n* = 10). Exact *p* values are indicated for each comparison ( ^∗^ *p* < 0.05;  ^∗∗^ *p* < 0.05). KD, knockdown.

Since *TAF1* plays a critical role in neuronal development, we investigated its potential involvement in epileptogenesis. To assess cellular excitability, we conducted whole‐cell patch‐clamp recordings on cultured cortical neurons after TAF1 knockdown (via shRNA) and compared them with control neurons. Ten neurons per group from three independent experiments were recorded. Key electrophysiological alterations were observed in TAF1‐deficient neurons. Neurons with TAF1 knockdown exhibited a significant depolarization (−48.7 ± 1.9 mV) compared with controls (−54.7 ± 1.76 mV; *p* < 0.05; Figure 5A,B). The firing frequency was markedly increased in neurons with TAF1 knockdown (16.8 ± 1.2 Hz vs. 11.6 ± 0.7 Hz in controls; *p* < 0.01; Figure 5C). On the other hand, no significant differences were detected in terms of action potential amplitude (TAF1 KD: 74.5 ± 5.6 mV vs. control: 81.7 ± 4.5 mV) or voltage threshold (TAF1 KD: −32.5 ± 1 mV vs. control: −29.0 ± 1.5 mV) (*p* > 0.05; Figure 5D,E). These findings suggest that TAF1 deficiency enhances neuronal excitability, providing a plausible cellular mechanism for the epileptic phenotype observed in patients with TAF1 mutations.

## 5. Discussion

TAF1 missense variants have emerged as an established cause of XLID with variable neurological involvement [5, 6]. The present study expands this clinical framework by identifying IESS as a novel, severe phenotypic manifestation and elucidating mechanistic pathways through which TAF1 deficiency disrupts neuronal development and excitability.

### 5.1. Phenotypic Expansion and Genotype–Phenotype Correlations

The epileptic encephalopathy observed in our patients represents a substantial departure from previously reported TAF1‐related phenotypes. Prior cohorts emphasized intellectual disability, dysmorphism, and movement disorders, with epilepsy—when present—manifesting as nonspecific seizure types in a minority subset [5, 6]. The occurrence of infantile spasms with hypsarrhythmia, the electroclinical hallmark of IESS, in two independent probands suggests that this represents a true phenotypic expansion rather than ascertainment bias. Both variants were maternally inherited, and segregation analysis confirmed that the asymptomatic mothers were heterozygous carriers. This is consistent with X‐linked recessive inheritance; skewed X‐inactivation may protect carrier females, as previously documented in TAF1‐related XLID [5].

The p.T79N variant resides within the N‐terminal TBP–interacting domain, disrupting a residue invariant across vertebrate orthologs (Figure 1C,D). This domain mediates TFIID core complex assembly; its perturbation would predictably compromise holoenzyme stability [2, 3]. The p.D1591N variant affects the tandem bromodomain, a specialized module for acetyl‐lysine recognition on histone tails that couples transcriptional machinery to chromatin state [10, 11]. Despite benign prediction by PolyPhen2—an algorithm with known limitations for variants in noncatalytic domains [12]—our functional data and evolutionary conservation support pathogenicity. The two variants reduced TAF1 protein levels to different extents, with p.T79N causing a greater reduction than p.D1591N, suggesting that the TBP‐interacting domain and the tandem bromodomain contribute differently to TAF1 folding or chaperone‐mediated quality control. Structural modeling analysis revealed that p.D1591N may affect TAF1 binding to DNA. These findings may explain the inconsistent clinical severity between the two cases, indicating that impaired DNA binding by TAF1 as a transcription factor results in a more severe phenotype. Notably, the magnitude of protein reduction measured in heterologous cells did not parallel clinical severity: Case 1 (p.T79N), whose variant caused the larger reduction in protein level, followed a milder clinical course than Case 2 (p.D1591N), indicating that residual TAF1 protein abundance alone cannot explain phenotypic severity. Rather, the location of the mutation and its specific functional consequence—particularly disruption of DNA‐binding capacity—appear to be critical determinants of clinical outcome.

To explore the submolecular basis of phenotypic variation, we reviewed previously reported TAF1 variants associated with epilepsy [5, 6, 13] and summarized their genetic locations, other phenotypes, and seizure phenotypes in Table S2. Several nonexclusive mechanisms may explain why TAF1 variants produce distinct phenotypic spectra (XLID, XDP, and IESS). First, variant location is likely important: variants in the N‐terminal TBP‐interacting domain may preferentially compromise TFIID holoenzyme assembly and basal transcription; bromodomain variants may selectively impair chromatin‐coupled transcription of activity‐regulated neuronal genes; and the XDP‐associated SVA retrotransposon insertion reduces the neuron‐specific isoform (nTAF1) predominantly in the striatum [4]. Second, the degree of residual TAF1 function may determine disease severity, as complete loss of TAF1 is embryonically lethal in model organisms [7], whereas hypomorphic variants with intermediate functional effects may permit survival with developmental and epileptic phenotypes. Third, tissue‐ and stage‐specific expression of TAF1 isoforms, together with genetic background, epigenetic modifiers, and X‐inactivation patterns in carrier females, likely shapes the regional and temporal vulnerability of the developing brain.

### 5.2. Transcriptional Dysregulation and Neuronal Development

RNA sequencing of TAF1‐deficient neuroblastoma cells revealed coordinated downregulation of gene networks governing nervous system development, neurogenesis, and axon guidance (Figure 3A). This transcriptional signature aligns with the established requirement for TFIID in activating cell type–specific enhancers during neuronal differentiation [14, 15]. Among validated targets, Draxin and Lgi1 warrant particular attention given their established roles in circuit assembly. Draxin functions as a netrin‐1 coreceptor, directing thalamocortical and commissural axon trajectories through DCC/UNC5 receptor complexes [16, 17]; its downregulation in TAF1‐deficient cells provides a mechanistic basis for the impaired neurite outgrowth we observed in primary cortical neurons (Figure 4). Lgi1, encoded by a gene recurrently mutated in autosomal dominant lateral temporal lobe epilepsy [18, 19], regulates synaptic AMPA receptor composition through interaction with ADAM22 and presynaptic Kv1.1 channels [20, 21]. The concordant reduction of Draxin, Lgi1, and voltage‐gated potassium channel subunits (Kcnn2 and Kcna4) in our transcriptomic data suggests that TAF1 coordinates a molecular program coupling structural maturation with ion channel expression.

We observed a selective neurite outgrowth defect—reduced axonal and total neurite length without altered dendritic spine density (Figure 4)—which is consistent with and extends previous observations in zebrafish and rat models [7, 8]. The selective vulnerability of axonal versus dendritic compartments may reflect differential dependence on TAF1‐regulated cytoskeletal or guidance programs, or alternatively, temporal dynamics of our culture system. Notably, Janakiraman et al. [8] reported reduced spontaneous excitatory postsynaptic currents in TAF1‐deficient rats attributable to presynaptic CaV3.1 downregulation, yet did not observe epileptic phenotypes. Our identification of complementary excitability mechanisms in a human cellular context resolves this apparent paradox and establishes epileptogenic potential.

### 5.3. Mechanisms of Hyperexcitability and Epileptogenesis

Whole‐cell patch‐clamp recordings demonstrated that TAF1 knockdown depolarizes resting membrane potential (−48.7 mV vs. −54.7 mV) and increases action potential firing frequency (16.8 Hz vs. 11.6 Hz) without altering voltage threshold or spike amplitude (Figure 5). These electrophysiological changes are consistent with reduced potassium conductance, as the Nernst equilibrium potential for K^+^ (−90 to −100 mV under our recording conditions) normally dominates resting potential determination. The coordinated downregulation of Kcnn2 (encoding KCa2.2/SK2, a small‐conductance calcium‐activated potassium channel critical for afterhyperpolarization and spike frequency adaptation) and Kcna4 (encoding Kv1.4, a rapidly inactivating A‐type channel governing repolarization and interspike interval) provides a plausible ionic basis for this phenotype [22, 23].

KCNN2 dysfunction has been directly implicated in human neurological disease: A gain‐of‐function variant causes tremulous myoclonus‐dystonia through altered striatal pacemaker activity [24], whereas pharmacological SK channel modulation influences seizure susceptibility in multiple models [25]. KCNA4 mutations cause a distinctive syndrome of striatal abnormality, congenital cataract, and intellectual disability, with electrophysiological studies confirming enhanced neuronal excitability [23]. The convergence of these channelopathies with our TAF1 findings suggests that transcriptional downregulation of potassium conductances represents a final common pathway for epileptogenesis in diverse genetic etiologies.

Reduced expression of *Lgi1* may constitute an additional, nonexclusive mechanism contributing to hyperexcitability. Beyond its structural role in axon guidance [26], Lgi1 forms a trans‐synaptic complex with ADAM22 and ADAM23 that stabilizes AMPA receptors and modulates Kv1.1‐mediated presynaptic repolarization [20, 21]. Autoantibodies against Lgi1 in limbic encephalitis disrupt this interaction, reducing synaptic AMPA receptors and producing faciobrachial dystonic seizures and temporal lobe epilepsy [21]. The 50% reduction in Lgi1 expression we observed in TAF1‐deficient cells, although incomplete, may suffice to perturb this finely tuned regulatory system, particularly during critical developmental windows when channel and receptor stoichiometries are established.

### 5.4. Limitations and Future Directions

Our conclusions derive from in vitro systems—heterologous overexpression in HEK293T cells, neuroblastoma transcriptomics, and murine primary cortical cultures—each with inherent constraints. The absence of TAF1 knock‐in animals reflecting human missense variants precludes assessment of developmental timing, brain region–specific vulnerability, and circuit‐level epileptogenesis. The divergent epilepsy outcomes of the two patients may also be related to the timing of treatment initiation: Case 1 received antiseizure therapy approximately X months after onset, whereas Case 2 started ACTH approximately 7 months after onset; early treatment has been associated with more favorable outcomes in IESS. The phenotypic severity disparity between our patients (seizure‐free at age 2 years vs. refractory spasms with profound disability) likely reflects variant‐specific functional effects, modifying genetic or epigenetic factors, or stochastic developmental events, none of which our current experiments can resolve. Future generation of patient‐specific induced pluripotent stem cell–derived neuronal models and, ultimately, targeted knock‐in mice will be essential to validate these mechanisms in vivo and explore therapeutic avenues—including potassium channel activators or Lgi1 pathway modulators—that our findings suggest. In addition, our electrophysiological analyses assessed intrinsic membrane properties and evoked firing but did not examine miniature excitatory or inhibitory postsynaptic currents (mEPSCs and mIPSCs); we therefore cannot determine the relative contributions of altered intrinsic excitability and imbalanced synaptic transmission. Notably, reduced spontaneous excitatory postsynaptic currents have been reported in Taf1‐deficient rat cerebellum [8]. Nevertheless, the depolarized resting membrane potential and increased evoked firing with unchanged voltage threshold and spike amplitude observed here support a primary contribution of enhanced intrinsic excitability, consistent with the transcriptional downregulation of potassium channel genes. Future studies incorporating synaptic physiology in TAF1‐deficient neurons will be needed to dissect these components.

## Author Contributions

Leilei Mao and Jing Peng contributed equally to this work and should be considered co‐corresponding authors.

## Funding

This study was supported by the National Natural Science Foundation of China (10.13039/501100001809) (82501748), Changsha Natural Science Foundation (kq2208383), and Natural Science Foundation of Hunan Province (10.13039/501100004735) (2024JJ678).

## Ethics Statement

This study was approved by the Ethics Committee of Xiangya Hospital of Central South University, China (Human Study/Protocol #201603205), and conducted following the ethical standards described by the Declaration of Helsinki. Written informed consent was obtained from parents.

## Consent

The authors have nothing to report.

## Conflicts of Interest

The authors declare no conflicts of interest.

## Supporting Information

Additional supporting information can be found online in the Supporting Information section.

## Supporting information


**Supporting Information 1** Figure S1: The structural modeling of the two TAF1 mutation sites; Figure S2: shRNA knockdown efficiency of Taf1 in Neuro‐2a cells.

**Supporting Information 2** Table S1: In silico prediction results of the two novel *TAF1* missense variants.

**Supporting Information 3** Table S2: Summary of previously reported *TAF1* variants associated with epilepsy.

## Data Availability

The data that support the findings of this study are available on request from the corresponding authors. The data are not publicly available due to privacy or ethical restrictions.
