# Supplementary material for: Infantile Epileptic Spasm Syndrome Caused by TAF1 Missense Variants: Expanding the Epileptic Phenotype and Revealing Underlying Impairments in Neuronal Excitability and Development
Source: Hum Mutat. 2026 Sep 16;2026:4104648. doi: 10.1155/humu/4104648 (PMC13583436; doi:10.1155/humu/4104648)
Supplement: Supplementary file 1 [file HUMU-2026-4104648-s001.docx]

**Table S1.** In silico prediction results of the two novel *TAF1* missense variants.

| Variant | Nucleotide change | Amino acid change | CADD | PolyPhen-2 | MutationTaster | LRT |
| --- | --- | --- | --- | --- | --- | --- |
| *TAF1*:c.236C>A | c.236C>A (NM_004606.4) | p.T79N | 23.7, Deleterious | 0.985, Probably damaging | Disease causing | Deleterious |
| *TAF1*:c.4771G>A | c.4771G>A (NM_004606.4) | p.D1591N | 22.4, Damaging | 0.014, Benign | Disease causing | Deleterious |

Note: CADD, Combined Annotation Dependent Depletion; LRT, Likelihood Ratio Test.
