# Supplementary material for: Infantile Epileptic Spasm Syndrome Caused by TAF1 Missense Variants: Expanding the Epileptic Phenotype and Revealing Underlying Impairments in Neuronal Excitability and Development
Source: Hum Mutat. 2026 Sep 16;2026:4104648. doi: 10.1155/humu/4104648 (PMC13583436; doi:10.1155/humu/4104648)
Supplement: Supplementary file 2 [file HUMU-2026-4104648-s002.docx]

**Novel missense variants of *TAF1* are associated with infantile epileptic spasms and impaired** **neuronal** **excitability and development**

Hui Xiao^1^, Changning Xie^1^, Pan Peng^1^, Xiaoyuan Ni^1^, Leilei Mao*^1,2^, Jing Peng*^1,3^

1 Department of Pediatrics, Xiangya Hospital, Central South University, Xiangya Road 87, Changsha, 410008, Hunan, China.

2 Department of Pediatrics, Xiangya Hospital, Central South University, Xiangya Road 87, Changsha, 410008, Hunan, China.329544100@qq.com.

3 Department of Pediatrics, Xiangya Hospital, Central South University, Xiangya Road 87, Changsha, 410008, Hunan, China. pengjing4346@163.com.

*These authors contributed equally to this work and should be considered co-corresponding authors.

**Supplementary material**

**Fig. S1** Structural modeling of the two TAF1 mutation sites. The green ribbon represents the crystal structure of TAF1, while the white ribbon represents the modeled structure. As shown in the figure, the white and green ribbons overlap well, indicating reliable modeling of the TAF1 structure. P.D1591N is located in the lower portion of the figure, close to DNA, and resides within the bromodomain (BRD) of kinase domain 2, which can interact with ASF1A and ASF1B (PMID: 12842904), though no structural resolution is available for this interaction. P.T79N is located in the upper portion of the figure, near kinase domain 1, which binds to the TATA-box binding protein (TBP); however, p.T79N itself faces outward and has no interaction.

**250KD**

**40KD**

**Fig. S2** shRNA knockdown efficiency in Neuro-2a cells. Western blotting showed that compared to control cells, the protein levels of the Taf1 protein were reduced in Neuro-2a cells by almost 70% after stable Taf1 knockdown. The TAF1 antibody (Cell Signaling Technology, Rabbit 1:1000) was used for measurement. Student’s t-test was applied to compare groups. All data are presented as mean ± standard error of the mean (SEM). The p-value was divided into three levels: *P < 0.05, **P < 0.005, ****P < 0.0001.
